# Aliskiren therapy in hypertension and cardiovascular disease: a systematic review and a meta-analysis

**DOI:** 10.18632/oncotarget.19382

**Published:** 2017-07-19

**Authors:** Shufang Fu, Xin Wen, Fei Han, Yin Long, Gaosi Xu

**Affiliations:** ^1^ Medical Center of The Graduate School, Nanchang University, Nanchang, China; ^2^ Grade 2013, School of Stomatology, Nanchang University, Nanchang, China; ^3^ Kidney Disease Center, The First Affiliated Hospital, College of Medicine, Zhejiang University, Hangzhou, China; ^4^ Grade 2013, The Second Clinical Medical College of Nanchang University, Nanchang, China; ^5^ Department of Nephrology, The Second Affiliated Hospital of Nanchang University, Nanchang, China

**Keywords:** aliskiren, hyperkalaemia, kidney injury, cardiovascular disease

## Abstract

The efficacy and safety of aliskiren combination therapy with angiotensin converting enzyme inhibitors (ACEIs) or angiotensin receptor blockers (ARBs) in patients with hypertension and cardiovascular disease remains attractive attention. We searched the Cochrane Central Register, the Clinical Trials Registry, EMBASE, MEDLINE and PubMed for relevant literatures up to January 2017. A total of 13 randomized controlled trials (RCTs) with 12222 patients were included in this study, and the combined results indicated that aliskiren in combination therapy with ACEIs or ARBs had remarkable effects in reducing systolic blood pressure (SBP) [weighted mean differences (WMD), -4.20; 95% confidential intervals (CI) -5.44 to -2.97; *I*^*2*^, 29.7%] and diastolic blood pressure (DBP: WMD, -2.09; 95% CI -2.90 to -1.27; *I*^*2*^, 0%) when compared with ACEIs or ARBs monotherapy, but with significantly increased the risk of hyperkalaemia [relative risk (RR), 1.45; 95% CI 1.28 to 1.64; *I*^*2*^,10.6 %] and kidney injury ( RR, 1.92; 95% CI 1.14 to 3.21; *I*^*2*^, 0%). Besides, there was no significant difference in the incidence of major cardiovascular events (RR, 0.95; 95% CI 0.89 to 1.02; *I*^*2*^, 0%) between the combined therapy and ACEIs or ARBs monotherapy. In conclusion, this meta-analysis demonstrated that aliskiren in combination therapy with ACEs/ARBs could control BP effectively, but is associated with increasing risks of hyperkalaemia and kidney injury, and have no benefit in preventing of major cardiovascular events.

## INTRODUCTION

Excessive renin-angiotensin-aldosterone system (RAAS) activity is a major underlying cause of many pathological states such as hypertension, heart failure, chronic renal failure and related cardiovascular disorders. [1] Because of Ang II generation and the inhibition of the RAAS is an effective way to intervene in the pathogenesis of these disorders. [2-4] RAAS inhibitors, such as angiotensin converting enzyme inhibitors (ACEIs) and angiotensin AT1- receptor blockers (ARBs) have proved to be highly successful treatments and become the conventional strategy in these populations.

However, drugs fail to absolutely block RAAS activity with increasing the concentration of renin because they attenuate the negative feedback effect of Ang II on renin release, and such high renin levels will activate the (pro)renin receptor ((P)RR). Once activated, PRR is able to trigger the catalytic activation of the inflammatory and profibrotic prorenin/PRR/MAP kinase/ ERK 1/2 cascade. Consequently, ERK 1/2 activation results in an overexpression of the genes coding for several mediators of the mesangial cells proliferation and sclerosis, such as the transforming growth factor-β1 (TGF- β1), the plasminogen activator inhibitor-1 (PAI-1), fibronectin and collagen, which lead to the progression of renal damage and cardiovascular disorders along with Ang II generation, which is called “RAAS escape’”. Aliskiren, a direct renin inhibitor (DRI), has no effect on (pro)renin binding to its receptors, but blocks the most critical site of renin activation, which demonstrated as a considerable reduction in plasma rennin activity (PRA), and with PRA decreases significantly, Ang I, Ang II and aldosterone also decrease significantly, [5] which seems contribute to a complete cardiorenal protection.

In the past several important large-scale trials of aliskiren therapy had been conducted to evaluate the potential cardiorenal effects on morbidity and mortality. The ALTITUDE trial suggested the addition of aliskiren to standard therapy with renin-angiotensin system blockades in patients with type 2 diabetes had no effect on cardiovascular and renal events, and it may even be harmful. [6] The ASTRONAUT trial concluded aliskiren plus standard therapy did not reduce the rate of cardiovascular (CV) death or heart failure (HF) rehospitalization among hospitalizations for heart failure (HHF) patients. [7] The AQUARIUS trial indicated that aliskiren therapy did not result in improvement or slowing of the progression of coronary atherosclerosis, and has no significant effect on the risk of major cardiovascular outcomes. [8] The ATMOSPHERE trial is currently evaluating the effects of an additional treatment of aliskiren in patients with HF and the result is that the addition of aliskiren to enalapril led to more adverse events without an increase in benefit. [9]

The previous meta-analysis provided discordant conclusions. Weir et al. and Zheng et al. suggested there was no statistically significant difference in hyperkalaemia between aliskiren combinatiom therapy and ACEIs or ARBs monotherapy in patients with essential hypertension. [10, 11] White et al. concluded that aliskiren combination therapy caused an added risk of hyperkalaemia but had no effect on renal dysfunction in essential hypertension. [12] While Harel Z et al. found that aliskiren combination therapy was related to the increased risk for hyperkalaemia, but had no effect on the acute kidney injury (AKI) in clinically diverse populations (different disease status). [13] Harel Z defined AKI as serum creatinine concentration > 176.8 umol/L or 2.0 mg/dL in their study, which might be incorrect according to the KIDGO Clinical practice guideline. The emergence of contradictory results may was due to two reasons, firstly, their research population is non -uniform; secondly, their data were too old to confer accurate and updated information. Moreover, they did not observe the efficacy of aliskiren therapy on cardiovascular events.

Considering these factors, it was thus necessary to conduct a new and comprehensive analysis including the latest published studies with large sample size.

## RESULTS

### Study enrolment and characteristics

**Figure 1:**
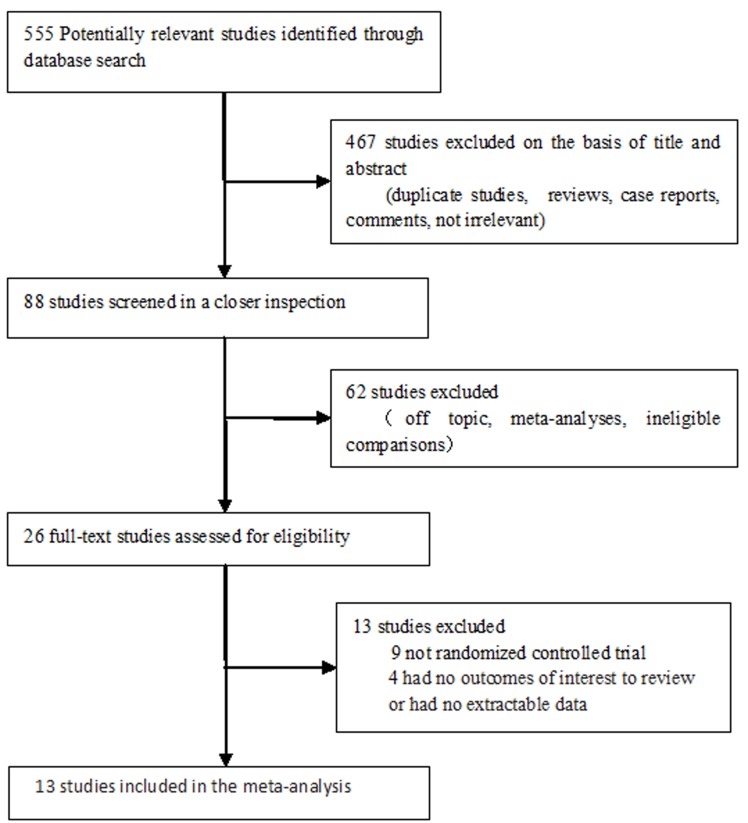
Flow diagram for the selection of studies inclusion in the meta-analysis

**Figure 2:**
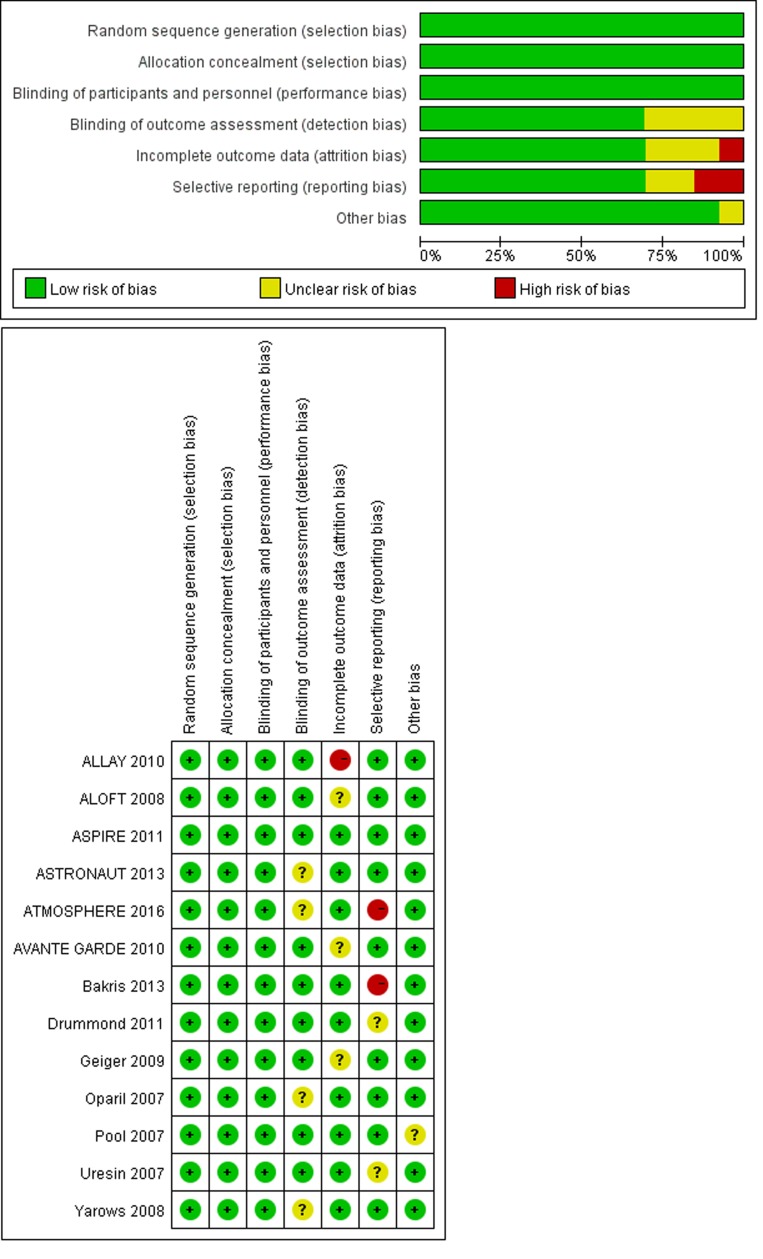
Risk of bias graph and risk of bias summary

We enrolled 13 studies with 12222 patients in the present analysis evaluated the efficacy and safety of aliskiren in combination therapy with ACEs/ARBs in patients with hypertension and cardiovascular disease, and Figure 1 showed the selection process. Ten studies involved in the analysis of hyperkalaemia, eight studies involved in kidney injury and four studies reported major cardiovascular events. The characteristics of the included studies and the risks of bias for RCTs were described in Table 1 and Figure 2, respectively.

**Table 1:** Characteristics of included studies

Study	Study duration	No of patients	Mean Age (year)	Study population	Diabetics (%)	GFR (ml/min)	Intervention	Control	The observed event
ALOFT 2008	12w	302	68	Hypertension with New York Heart Association class II to IV heart failure	31;30	70;68	Aliskiren 150mg plus stable dose of ACEIs or ARBs	Placebo plus stable dose of ACEIs or ARBs	The hyperkalaemia and kidney injury
ASTRONAUT 2013	6m	1350	61	Patients hospitalized for HF with reduced LVEF,	NA;NA	NA;NA	Aliskiren 300mg plus ACEIs or ARBs	Placebo plus ACEIs or ARBs	Cardiovascular (CV) death or HF rehospitalization among HHF patients.
ALLAY 2010	36w	306	59	Hypertension with left ventricular hypertrophy	27;22	83;85	Aliskiren 300mg plus losartan 100mg	losartan100 mg	The hyperkalaemia and kidney injury
AVANTE GARDE 2010	8w	550	63	Acute coronary syndrome without LEVF <50%	21;21	75;74	Aliskiren 300mg plus valsartan 320mg	valsartan320mg	The hyperkalaemia and kidney injury; CV death and myocardial infarction
ASPIRE 2011	36w	820	60	AMI with LEVF <45%	23;22	80;81	Aliskiren 300mg plus ACEIs or ARBs	Placebo plus ACEIs or ARBs	The hyperkalaemia and kidney injury; composite of CV death
Drummond 2011	12w	363	56	Hypertensive Diabetes	100;100	NA;NA	Aliskiren 300mg plus valsartan 160mg	Placebo plus Valsartan 160mg	The changes in msDBP and msSBP; the hyperkalaemia and kidney injury
Bakris 2013	8w	1143	55	Hypertension with type2 diabetes	100;100	95;95	Aliskiren 300mg plus valsartan 320mg	valsartan 320mg	The change in Ambulatory blood pressure; the hyperkalaemia
ATMOSPHERE 2016	36.6m	4676	63	CHF with LEVF<30%	28;28	74;74	Aliskiren 300mg plus Enalapril 10mg	Enalapril 10mg	The death from cardiovascular causes or hospitalization for heart failure.
Oparil 2007	8w	906	52	Stage I-II hypertension	NA;NA	NA;NA	Aliskiren 300mg plus valsartan 320mg	valsartan 320mg	The changes in msDBP and msSBP; the hyperkalaemia and kidney injury
Pool 2007	8w	355	57	Mild to moderate hypertension	11;7	NA;NA	Aliskiren 300mg plus valsartan 320mg	Valsartan320mg	The changes in msDBP and msSBP
Uresin 2007	8w	555	60	Stage I-II hypertension with Diabetes mellitus type 1or 2	100;100	NA;NA	Aliskiren 300mg plus ramipril 10mg	Ramipril 10mg	The hyperkalaemia and kidney injury
Geiger 2009	8w	620	53	Hypertension	11;12	NA;NA	Aliskiren 300mg plus valsartan 320mg	Valsartan 320mg	The changes in msDBP and msSBP
Yarows 2008	8w	276	57	Stage 2 Hypertension	NA;NA	NA;NA	Aliskiren 300mg plus valsartan 320mg	Valsartan 320mg	The changes in msDBP and msSBP

Abbreviations: ACEIs or ARBs: angiotensin converting enzyme inhibitors or angiotensin receptor blockers; msSBP: mean sitting systolic BP; msDBP: mean sitting diastolic BP; GFR: glomerular filtration rate; CV death: Cardiovascular death; HF: heart failure; HHF: hospitalizations for heart failure; CHF: chronic heart failure; LEVF: left ventricular ejection fraction; m: month; w: week; NA: not available

In columns "diabetes" and "GFR" showed a series of values (e.g. 31; 30 or 70; 68), the former value is related to Intervention, and the later value is related to Control

### Meta-analysis results

### The primary outcomes

### Antihypertension effects

**Figure 3:**
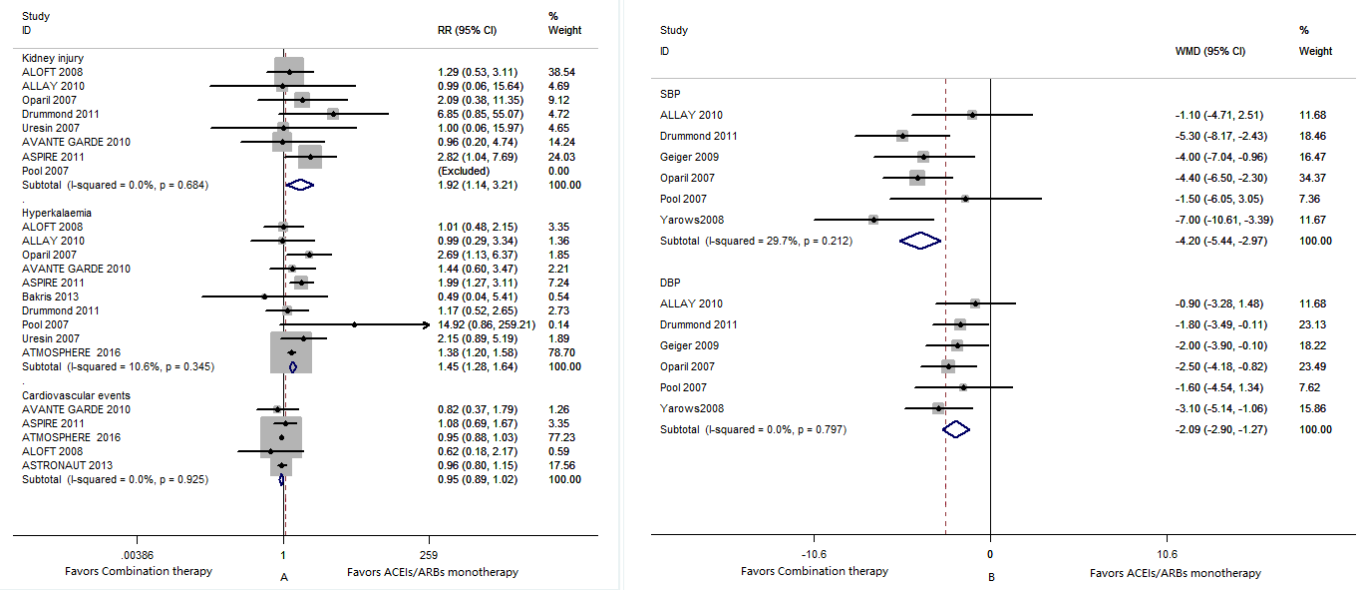
A: Forest plot shows the risk of secondary outcomes in aliskiren combonation therapy or ACEIs or ARBs monotherapy; B: Comparison of the effect of antihypertension between aliskiren combination therapy and ACEIs or ARBs monotherapy Abbreviations: kidney injury; ACEIs or ARBs: angiotensin converting enzyme inhibitors or angiotensin receptor blockers; SBP: systolic blood pressure; DBP: diastoblic blood pressure

Six trials [14-19] involved in the analysis of antihypertension and the result suggested aliskiren in combination therapy with ACEs/ARBs was superior to ACEI or ARB monotherapy in SBP reduction (WMD, -4.20; 95% CI -5.44 to -2.97; *I*^*2*^, 29.7%) and DBP reduction (WMD, -2.09; 95% CI -2.90 to -1.27; *I*^*2*^, 0%) as described in Figure 3B.

### The secondary outcomes

#### Hyperkalaemia and subgroup analysis

**Figure 4:**
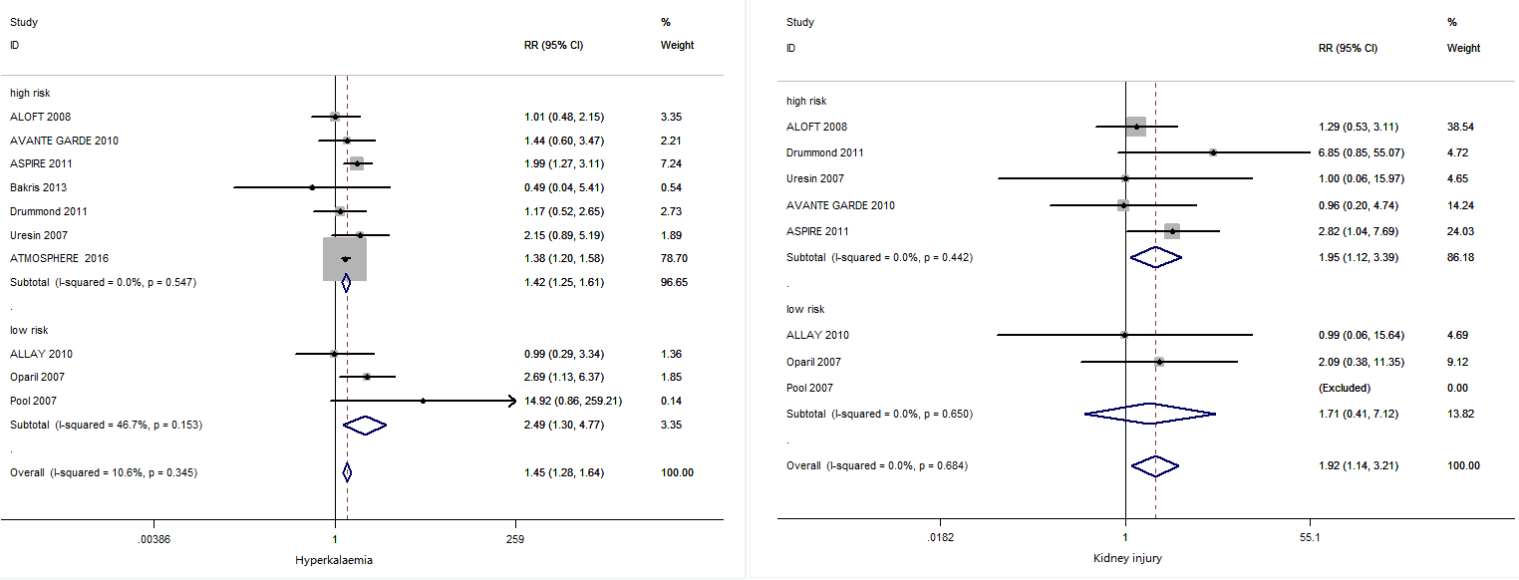
Subgroup analyses of hyperkalaemia and kidney injury in high risk and low risk groups

Ten studies [9, 14, 15, 17, 19-24] reported on the outcome of hyperkalaemia (Table 2 and Figure 3A). Combination therapy was significantly associated with the risk of hyperkalaemia compared with an ACEI or an ARB monotherapy (RR 1.45; 95% CI 1.28 to 1.64; *I*^*2*^, 10.6 %). Among ten studies involved in hyperkalaemia, seven studies [9, 14, 20-24] enrolled patients at high risk and three [15, 17, 19] enrolled patients at low risk, meta-analysis found aliskiren combination therapy did significantly increase the risk for hyperkalaemia in both groups: RR in high risk patients 1.42 (95% CI 1.25 to 1.61) and in low risk patients 2.49 (95% CI 1.30 to 4.77), as showed in Figure 4.

**Table 2:** Secondary outcomes about aliskiren combination therapy or ACEIs/ARBs monotherapy

Study	Hyperkalaemia		Kidney injury		Cardiovascular events	
	Combination	ACEI/ARB	Combination	ACEI/ARB	Combination	ACEI/ARB
ALLAY 2010	5/154	5/152	1/154	1/152	NA	NA
ALOFT 2008	13/156	12/146	11/156	8/146	4/156	6/146
ASPIRE 2011	55/423	26/397	15/423	5/397	39/422	34/397
AVANTE GARDE 2010	12/279	8/268	3/279	3/268	11/278	13/268
ASTRONAUT 2013	NA	NA	NA	NA	178/674	182/686
Drummond 2011	12/181	10/177	7/181	1/177	NA	NA
Bakris 2013	1/574	2/565	NA	NA	NA	NA
ATMOSPHERE 2016	401/2340	291/2336	NA	NA	770/2340	808/2336
Oparil 2007	18/446	7/455	2/446	2/455	NA	NA
Pool 2007	7/178	0/177	0/178	0/177	NA	NA
Uresin 2007	15/277	7/278	1/277	1/278	NA	NA

Abbreviations: ACEIs or ARBs: angiotensin converting enzyme inhibitors or angiotensin receptor blockers; msSBP: mean sitting systolic BP; msDBP: mean sitting diastolic BP; GFR: glomerular filtration rate; CV death: Cardiovascular death; CHF: chronic heart failure; LEVF: left ventricular ejection fraction; m: month; w: week; NA: not available

#### Kidney injury and subgroup analysis

Eight studies [14, 15, 17, 19, 20, 22-24] involved in kidney injury (Table 2 and Figure 3A). Combination therapy was markedly associated with the risk of kidney injury compared with an ACEI or an ARB monotherapy (RR, 1.92; 95% CI 1.14 to 3.21; *I*^*2*^, 0%). Among eight studies involved in kidney injury, five studies [14, 20, 22-24] enrolled patients at high risk and three [15, 17, 19] enrolled patients at low risk, meta-analysis found aliskiren combination therapy did not significantly increase the risk for kidney injury in low risk group but did significantly increase in high risk group: RR in high risk patients 1.95 (95% CI 1.12 to 3.39) and in low risk patients 1.71(95% CI 0.41 to 7.12), as showed in Figure 4.

#### Major cardiovascular events

Five studies [7, 9, 20, 22, 23] involved in major cardiovascular events (Table 2 and Figure 3A). There was no statistically significant difference in major cardiovascular events between the two groups (RR, 0.95; 95% CI 0.89 to 1.02; *I*^*2*^, 0%).

### Sensitivity analysis and publication bias

**Figure 5:**
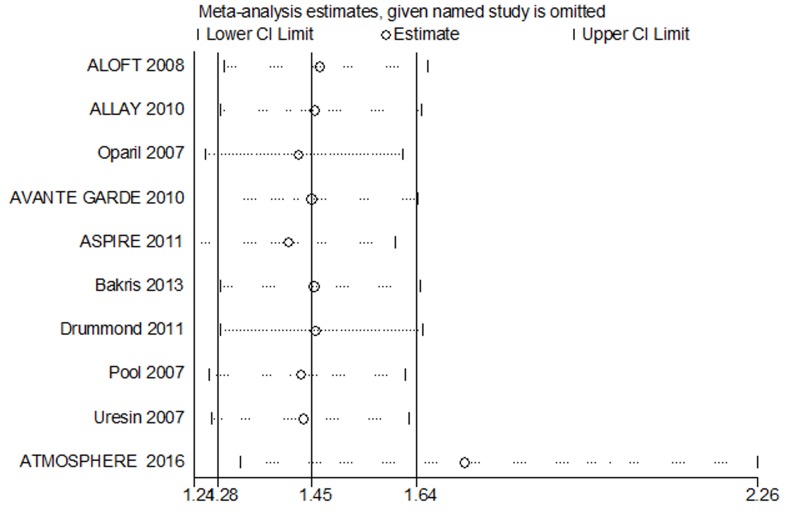
Sensitivity analysis of hyperkalaemia in patients with hypertension and cardiovascular disease

In our analysis, we performed a sensitivity analysis to seek for the source of heterogeneity. There was a significant effect on the result of the RR and 95% CI (Figure 5), which showed that the ATMOSPHERE study may be the source of heterogeneity in hyperkalaemia, but the heterogeneity was low (*I*^*2*^, 10.6 %) and when the ATMOSPHERE study was excluded, there was no effects on the statistical significance. The Egger’s test and Begg’s funnel plot showed no evidence of publication bias for the primary outcomes and secondary outcomes.

## DISCUSSION

The efficacy and safety of aliskiren has been always the focus of attention, especially when used in patients prescribed ACEIs or ARBs. The results of ASPIRE, ATMOSPHERE, ALTITUDE and ASTRONAUT suggested that such aliskiren combination therapy failed to reduce mortality and was associated with an excessive risk of adverse events, such as hyperkalaemia, hypotension, and renal failure.

RAAS inhibitors are capable of blocking aldosterone secretion from the adrenal gland because of blocking Ang II generation. Aldosterone, acting on sodium retention and urinary potassium excretion in response to increases in ambient angiotensin and extra cellular potassium concentration, thus ACEIs, ARBs and aliskiren are capable of causing hyperkalaemia. [25] In addition, RAAS blockers also influence renal hemodynamic primarily through dilation of the efferent arteriole, which lead to reduced intraglomerular pressure [26] and the decline in glomerular filtration rate (GFR), such decreases in GFR are manifested by an increase in serum creatinine levels. It seems that each class of RAAS blockers can cause hyperkalaemia and kidney injury, but this is not the case.

However, RAAS inhibitors might be expected to result in an increased incidence of hyperkalemia and kidney injury in susceptible populations such as CKD, HF and diabetes mellitus. Cause these susceptible populations have an impaired capacity for renal potassium excretion and are therefore at increased risk of developing hyperkalaemia. Moreover, in such patients, a compensatory increase in efferent, relative to afferent, arteriolar resistance occurs, which serves to increase or maintain GFR in single nephron. [25, 27] However, in patients with CKD or HF, blockade of angiotensin II production or action interferes with this compensatory response, resulting in a decrease in glomerular capillary pressure and, consequently, a reduction in GFR. [25] To be concluded, treatment of patients with failing kidneys or HF with ACEIs, ARBs or aliskiren might therefore not only cause hyperkalemia but can also cause kidney injury.

In analyses of Weir et al. White et al. and Zheng et al. enrolled patients with essential hypertension without cardiovascular disease or renal insufficiency at baseline, which might be representative of a lower-risk population. In contrast, Harel Z et al. included population with a broad range of baseline characteristics including lower-risk populations (essential hypertension without CKD or heart failure) and higher-risk populations (CKD, heart disease, diabetes mellitus). In our article, we included 13 trials with a total population of 12222 patients, and the study population consisted of hypertension and cardiovascular diseases (such as HF and coronary disease), as described in Table 1. Because there was no heterogeneity in our outcomes (hyperkalaemia: *I*^*2*^, 10.6 %; kidney injury: *I*^*2*^, 0%; major cardiovascular events: *I*^*2*^, 0%), and we have attempted to perform subgroup analysis to study the influence of disease types on the heterogeneity, we found that the heterogeneity would increase in both groups. Therefore we abruptly regard them as a study population in case of data loss and result bias.

According to the results of our meta-analysis, aliskiren combination therapy could control BP effectively, but with increasing risks of hyperkalaemia and kidney injury. Compared to ACEIs/ARBs monotherapy, the dual blockade of aliskirien combination therapy cause the more significant decrease in aldosterone due to the more significant blockade of Ang II generation, which should not only account for the superior antihypertension effect, but also the increased incidence of hyperkalemia.

Because the incidence of hyperkalaemia and kidney injury may be linked with the susceptible populations (high-risk populations), we stratified patients by their risk of hyperkalaemia and kidney injury into low and high risk groups and conducted subgroup analyses to discuss the effects of susceptible and non-susceptible populations. Aliskiren combination therapy did significantly increase the risk for hyperkalaemia in both groups but aliskiren combination therapy did not significantly increase the risk for kidney injury in low risk group but did significantly increase in high risk group when compared with ACEIs or ARBs. So we should take caution in combining aliskiren with an ACE inhibitor or an ARB, particularly in patients with heart disease, CKD and diabetes mellitus.

Aliskiren has been shown to be effective in lowering BP and affect proteinuria, left ventricular mass index and brain natriuretic peptide (BNP) levels in patients with albuminuria, LVH and HF when in combination with ACEs/ARBs, [15, 20, 28, 29] and these factors are also associated with the increased risk of cardiovascular diseases. Interestly, there was no statistically significant difference in the incidence of major cardiovascular events, in other words, aliskiren combination therapy fail to provide more effect on cardiovascular protection, maybe the overwhelming advantage on reducing BP, proteinuria and BNP in aliskiren combination therapy, was offset by potential side effects. [7]

There are several limitations in our study. We didn’t analyze the effects of dosage, duration of treatment and other antihypertension drugs on the results. Therefore for future study, the role of treatment duration and dosage should be taken into consideration before evaluating clinical outcomes.

In conclusion, this meta-analysis revealed that the use of aliskiren add-on therapy could control BP effectively but with increasing risk of hyperkalaemia and kidney injury, and have no benefits in preventing of cardiovascular events among patients with hypertension and cardiovascular disease prescribed conventional therapy with ACEIs or ARBs.

## MATERIALS AND METHODS

### Participants, interventions and outcome measurements

We included studies that evaluated the efficacy and safety of aliskiren in combination therapy with ACEs/ARBs in patients with hypertension and heart failure (with a history of hypertension). In these articles, hyperkalaemia was defined as a serum potassium level of > 5.5 mmol/L, and 8 out of 13 studies reported this outcome related to “a serum Creatinine concentration > 2,0 mg/dl”, the span of study duration in the 8 studies is very large (varying from 8 weeks to 36 weeks), according to the KIDGO Clinical practice guideline, “a serum Creatinine concentration > 2,0 mg/dl” cannot defined as Acute Kidney Diease (AKD), Chronic Kidney Diease (CKD) or Acute Kidney Injury (AKI), so kidney injury was abruptly defined as serum creatinine concentration > 176.8 umol/L or 2.0 mg/dL, and major cardiovascular events consisted of acute myocardial infarction (AMI), stroke, resuscitated cardiac arrest, death from cardiovascular cause, and hospitalization for HF. The primary outcome was the antihypertensive effects, and the secondary outcomes were the risks of hyperkalaemia, kidney injury and major cardiovascular events.

### Searching strategies

We searched the Cochrane Central Register, the Clinical Trials Registry, EMBASE, MEDLINE and PubMed for relevant literatures to Juanary 2017. Keywords include aliskiren or renin inhibitor or RAAS, hypertension, randomized controlled trial or RCT. Besides we reviewed the related research references.

### Inclusion and exclusion criteria

The inclusion criteria were as follows: (a) adults with hypertension and cardiovascular disease, (b) the study design should be a RCT, (c) the study described the efficacy and safety of aliskiren combination with either ACEIs or ARBs relative to an ACEI or ARB monotherapy that provided exact value on BP reduction, or the incidence of hyperkalaemia, kidney injury or cardiovascular outcomes, (d) complete data available to calculate relative ratio (RR) or weighted difference (WMD) with 95% confidence interval (CI), and (e) all dosing regimens of aliskiren, ACEIs or ARBs were considered. Exclusion criteria were as follows: (a) data from the studies could not be extracted and analyzed, (b) duplicate publications, and (c) non-human experimental studies.

### Study selection and data extraction

Three investigators (Shufang Fu, Xin Wen, Fei Han and Yin Long) independently performed the study selection. All the disagreements were resolved by discussion. We extracted the following data from each article: first author or study group name, year of publication, number of patients, study duration, the proportion of diabetes, the Mean and standard deviation (SD) value in systolic blood pressure (SBP) and diastolic blood pressure (DBP) reduction, the occurrence rate of hyperkalaemia, kidney injury or major cardiovascular outcomes.

### Assessment of the risk of bias

The risk of bias assessment tool by the Cochrane Collaboration was applied specifying the following bias domains: selection, performance, attrition, reporting bias and a judgment of low risk, unclear risk, or high risk was provided for each domain. [30]

### Statistical analysis

Statistical analysis was performed by Review Manager (version 5.3) and STATA statistical software (version 12.0). The adjusted estimated effects expressed as WMD or RR with 95% CI were calculated for continuous and dichotomous data, respectively, and summarized by Forest plots. Heterogeneity among studies was estimated by Cochrane’s Q-statistic and *I*^*2*^ tests. A random-effect model was used when Q-test exhibits a *P* < 0.05 or *I*^*2*^ test shows > 50%; otherwise, the fixed-effect model was selected. We performed subgroup analyses to assess for clinical heterogeneity stemming from the disease states, and we stratified patients by their risk of hyperkalemia and kidney injury into low and high risk groups, low risk was defined as patient without CKD, CAD, HF or diabetes mellitus. Inversely in high risk. Sensitivity analyses were conducted in the meta-analysis to examine the influence of an individual study. Publication bias was assessed by constructing a funnel plot and using Egger’s and Begg’s tests. A significant two-way *P* value for comparison was defined as *P* < 0.05.

## Abbreviations

ACEIs or ARBs: angiotensin converting enzyme inhibitors or angiotensin receptor blockers
msSBP: mean sitting systolic BP
msDBP: mean sitting diastolic BP
GFR: glomerular filtration rate
CV death: Cardiovascular death
HF: heart failure
HHF: hospitalizations for heart failure
CHF: chronic heart failure
LEVF: left ventricular ejection fraction
m: month
w: week
NA: not available
