# Correction: Aliskiren therapy in hypertension and cardiovascular disease: a systematic review and a meta-analysis

**DOI:** 10.18632/oncotarget.24255

**Published:** 2018-01-15

**Authors:** Shufang Fu, Xin Wen, Fei Han, Yin Long, Gaosi Xu

**Affiliations:** ^1^ Department of Nephrology, The Second Affiliated Hospital of Nanchang University, Nanchang, China; ^2^ Grade 2013, School of Stomatology, Nanchang University, Nanchang, China; ^3^ Kidney Disease Center, The First Affiliated Hospital, College of Medicine, Zhejiang University, Hangzhou, China; ^4^ Grade 2013, The Second Clinical Medical College of Nanchang University, Nanchang, China

This article has been corrected: Dr. Fu and Dr. Xu citations have been changed to affiliation 1 and affiliation 5 is now deleted.

Original article: Oncotarget. 2017; 8:89364-89374. https://doi.org/10.18632/oncotarget.19382

